# Two Different Tumors and Lung Aspergilloma: An Uncommon Etiopathogenic Association

**DOI:** 10.3390/medicina60060953

**Published:** 2024-06-07

**Authors:** Vlad Alexandru Ionescu, Gina Gheorghe, Cosmin Adrian, Alexandru Bebliuc, Cezar Pavelescu, Valentin Enache, Florentina Gheorghe, Nicolae Bacalbasa, Camelia Cristina Diaconu

**Affiliations:** 1 Faculty of Medicine, University of Medicine and Pharmacy Carol Davila Bucharest, 050474 Bucharest, Romania; vladalexandru.ionescu92@gmail.com; 2 Department of Internal Medicine, Clinical Emergency Hospital of Bucharest, 105402 Bucharest, Romania; 3 Department of Radiology, Clinical Emergency Hospital of Bucharest, 105402 Bucharest, Romania; 18.adriancosmin@gmail.com; 4 Department of Thoracic Surgery, Clinical Emergency Hospital of Bucharest, 105402 Bucharest, Romania; bebliuc@gmail.com (A.B.); cezarpavelescu@hotmail.com (C.P.); 5 Department of Anatomical Pathology, Clinical Emergency Hospital of Bucharest, 105402 Bucharest, Romania; valienache00@gmail.com; 6 Department of Biochemistry, Synevo Romania, 077040 Chiajna, Romania; florentina.9412@gmail.com; 7 Department of Surgery, University of Medicine and Pharmacy Carol Davila Bucharest, 050474 Bucharest, Romania; nicolae.bacalbasa@umfcd.ro; 8 Department of Surgery, Center of Excellence in Translational Medicine, Fundeni Clinical Institute, 022328 Bucharest, Romania; 9 Academy of Romanian Scientists, 050045 Bucharest, Romania

**Keywords:** adenocarcinoma, typical carcinoid, aspergilloma, etiopathogenesis, diagnosis, prognosis

## Abstract

Several cases reported in the literature have confirmed the link between pulmonary aspergillosis and various malignant diseases. Furthermore, it has been observed that the correlation between carcinoid tumor and lung adenocarcinoma is quite uncommon. The etiopathogenic mechanisms underlying these correlations remain poorly defined. We present the case of a patient with three of these diseases: a lung adenocarcinoma with a lepidic pattern, a typical carcinoid, and pulmonary aspergillosis. An additional noteworthy aspect of this case pertains to the timely detection of both lung malignancies. Thus, the necessity for further investigation to ascertain the pathogenic connection among the three diseases is underscored. The ultimate objective is to enhance the prognosis of individuals diagnosed with lung cancer, which is a prevailing malignant disease on a global scale.

## 1. Introduction

Pulmonary carcinoid tumors are considered rare neoplasms, with variable incidence rates ranging from 0.2 to 2 cases per 100,000 individuals in both the United States of America and Europe [1]. They represent 20–30% of all carcinoid tumors and 1–2% of all malignant lung tumors [1,2,3]. However, over the last four decades, the incidence of carcinoid tumors has increased by about 6% per year, regardless of demographic factors like age, gender, or race [4]. The rising frequency of carcinoid tumors can be attributed to the increased use of immunohistochemistry and improved diagnostic rates [1]. Their prevalence is marginally higher among women than men and among white people in comparison to black, Hispanic, or Asian individuals [1]. These neoplasms typically occur between the ages of 45 and 55 [1]. Pulmonary carcinoid tumors are often sporadic lesions, with familial occurrences being unusual [5]. Approximately 5% of patients with multiple endocrine neoplasia type 1 (MEN-1) develop carcinoid tumors [6,7].

The association of lung carcinoid tumors with non-small-cell lung cancer (NSCLC) is extremely infrequent [2]. After analyzing the data from the literature, only 11 cases of the two types of malignancies were identified [2]. Out of the total number of patients, four had adenocarcinomas associated with typical carcinoid tumors (TCs), two had squamous carcinomas associated with TCs, four had atypical carcinoid tumors (ACs) associated with adenocarcinomas, and one had an AC associated with a squamous carcinoma [2]. In each case, the tumors were found to be localized exclusively at the pulmonary level [2]. The pathophysiology of this connection between cancerous conditions is not well understood. Lung carcinoids arise from neuroendocrine cells, whereas primary lung adenocarcinoma originates from type II alveolar cells, derived from bronchioalveolar stem cells [8]. Consequently, the development of the two types of tumors may vary [2]. Conversely, Olofson et al. postulated that these tumors might arise from a shared precursor cell, which is associated with a BRAF mutation [9]. The veracity of this concept is supported by the fact that either bronchioloalveolar stem cells or pulmonary neuroendocrine cells originate from epithelial progenitor cells [8].

Aspergillus has the potential to induce a diverse range of lung diseases, which are contingent upon the patient’s immunological status and the preexisting lung condition [10]. The occurrence of lung carcinoid tumors associated with Aspergillus infection is uncommon [3]. To date, the specialized literature has documented a minimum of 40 cases of lung carcinomas that have been linked to the colonization of Aspergillus [11]. This pathogenic relationship can be explained by the presence of an underlying bronchial obstruction or the malignant transformation of cells originating from the structure of a preexisting cavity [11]. The diagnosis is difficult in these circumstances and may result in suboptimal treatment, which has an adverse effect on the prognosis of the patient.

We will present the case of an elderly female patient whose histological examination revealed the existence of pulmonary aspergilloma associated with both a carcinoid tumor and an adenocarcinoma, both situated inside the same lung lobe.

## 2. Case Report

A 75-year-old female, non-smoker, with no occupational exposure, presented to the emergency room with frequent episodes of hemoptysis, which began about a week before presentation. The patient also reported having had a dry cough during the previous three months. Her medical history included arterial hypertension grade II, chronic heart failure class II (New York Heart Association (NYHA)) with preserved left ventricular ejection fraction, mild aortic stenosis, mild aortic regurgitation, moderate mitral regurgitation, and dyslipidemia. The patient denied any significant family medical history. On clinical examination, she had normal weight, was afebrile, and had no peripheral edema. The superficial lymph node system was not palpable. The clinical assessment of the respiratory system indicated the presence of vesicular murmurs on both sides, with infrequent crackles in the lower part of the left hemithorax. The oxygen saturation level in the surrounding air was 94%. During the assessment of the cardiovascular system, we detected rhythmic heart sounds, with a systolic murmur in the aortic area that extended to the carotid arteries and a systolic murmur in the mitral area, irradiating to the axilla. The patient’s blood pressure was 140/90 mmHg, and the heart rate was 80 beats per minute. The abdomen was painless, both spontaneously and at palpation; intestinal transit and diuresis were normal. The patient was on long-term treatment with bisoprolol, perindopril, indapamide, acetylsalicylic acid, and atorvastatin. The initial biological evaluation did not identify significant pathological changes. A real-time polymerase chain reaction (RT-PCR) assay was conducted to detect the presence of SARS-CoV-2 infection, yielding a negative result. The electrocardiogram (ECG) revealed sinus rhythm with a heart rate of 80 beats per minute, a normal QRS axis, Q waves in the DII, DIII, and aVF derivations, and no alterations in the ventricular repolarization phase. Pleural or pulmonary lesions could not be identified by a chest X-ray.

**Figure 1:**
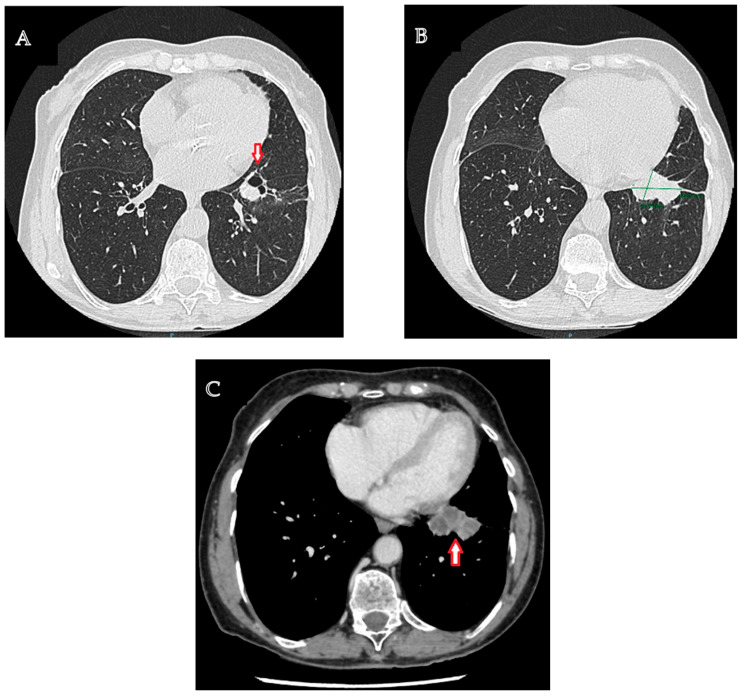
Typical carcinoid tumor located in the lower left lobe. (**A**). Contrast-enhanced chest computed tomography: a multiseptate lung tumor with multicystic appearance, located in the lower left lobe, associated with bronchial dilatations and discrete ground-glass areas. (**B**). Contrast-enhanced chest computed tomography: a lung tumor with maximum dimensions of 41/24/30 mm located in the lower left lobe. (**C**). Contrast-enhanced chest computed tomography, venous phase: a multiseptate lung tumor with multicystic appearance, located in the lower left lobe.

**Figure 2:**
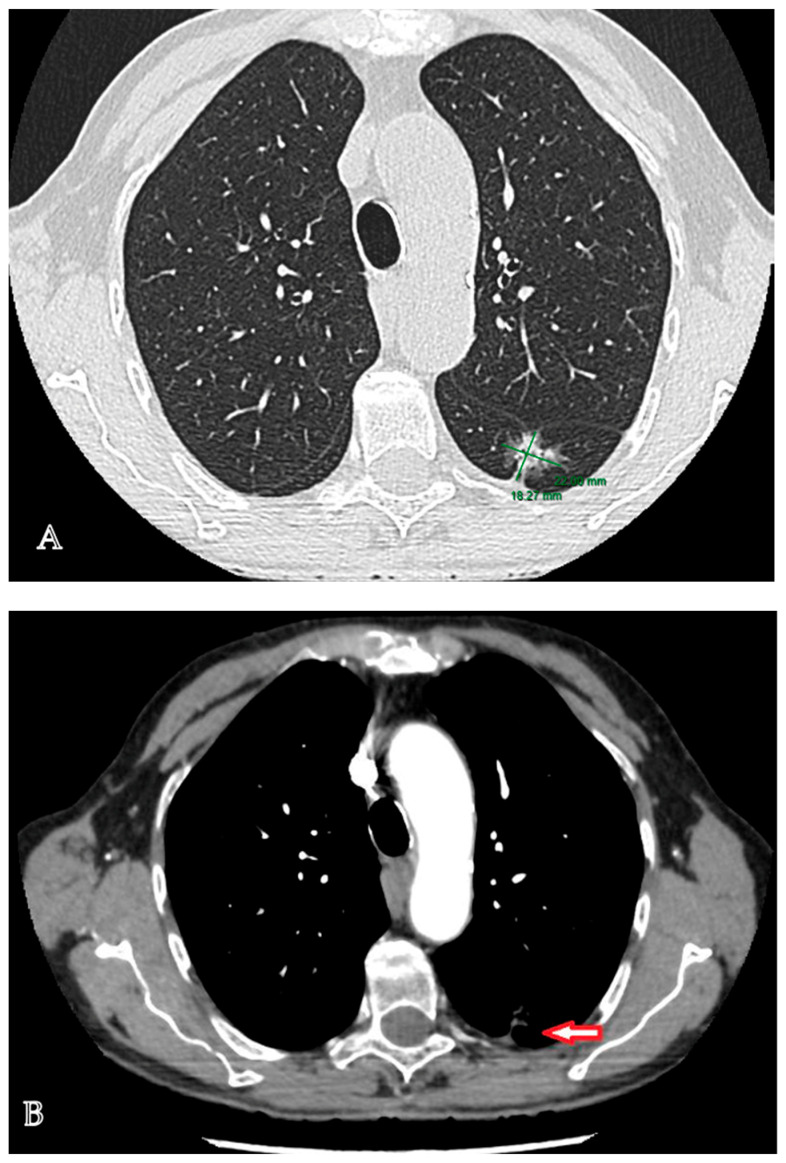
Lung adenocarcinoma located at the level of the left Fowler segment. (**A**). Contrast-enhanced chest computed tomography: a partially solid spiculiform tumor with maximum dimensions of 22/18/20 mm, located in the left Fowler segment. (**B**). Contrast-enhanced chest computed tomography, arterial phase: a partially solid spiculiform tumor that sends extensions to the parietal pleura and oblique fissure, exerting a retractive effect on both structures.

The diagnostic management was continued with a contrast-enhanced computed tomographic (CT) examination of the chest that highlighted a multiseptate lung tumor with multicystic appearance and peripheral gadolinophilia, with maximum dimensions of 41/24/30 mm, located in the lower left lobe, with direct communication to the bronchial lumen, associated with bronchial dilatations and discrete ground-glass areas (Figure 1A–C). In addition, at the level of the left Fowler segment, a partially solid spiculiform image with maximum dimensions of 22/18/20 mm was discovered, with extensions to the parietal pleura and oblique fissure, exerting a retractive effect on both structures (Figure 2A,B). Notably, mediastinal adenopathies were not identified. The suspicion of pulmonary cancer was raised with regard to the second lesion that was previously described.

Bronchoscopy revealed normal trachea and carina, a right bronchial tree without proliferative elements in the endoscopically approachable area, and a left bronchial tree without proliferative elements but with sero-hematous secretions at the level of a branch corresponding to the anterior segment of the left lower lobe.

We assessed the feasibility of conducting the biopsy under CT guidance, given the lack of a positive diagnosis. During this time, the patient experienced a significant hemoptypic event. The CT examination of the chest was performed in an emergency, revealing a dimensional growth of the two pulmonary lesions. Additionally, contrast-enhanced cerebral and abdominal–pelvic CT scans were performed, which revealed the absence of metastases. The decision for surgical intervention was made.

During the surgical procedure, a large, rigid lesion was identified in the basal pyramid, making limited resection impossible. Another lesion with spiculiform characteristics to palpation was discovered in the lower apical segment, as well as various anatomical vascular variations and extensive adenopathies with an inflammatory appearance. Under these circumstances, a left lower lobectomy with lymphadenectomy was performed, and the specimen was sent for histopathological examination. The postoperative evolution was good; however, imaging assessment revealed an inadequately enlarged lung with retracted mediastinum and hemidiaphragm. The patient was discharged with a pleural drainage rate of less than 100 mL per 24 h.

**Figure 3:**
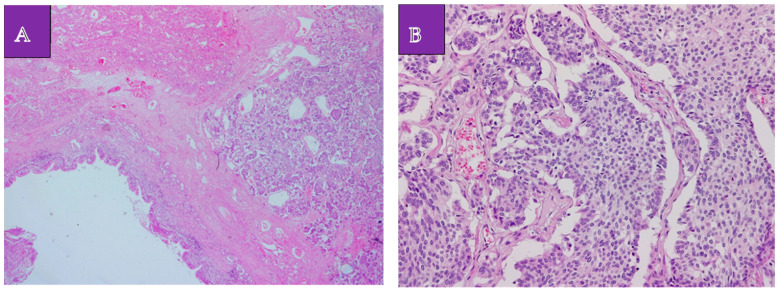
Carcinoid tumor proliferation. (**A**). Hematoxylin eosin, 50×. Bronchial tissue, tumor parenchyma, and carcinoid tumor proliferation. (**B**). Hematoxylin eosin 200×. Carcinoid tumor proliferation: neuroendocrine monoform cells of variable size, arranged in solid cellular aggregates.

**Figure 4:**
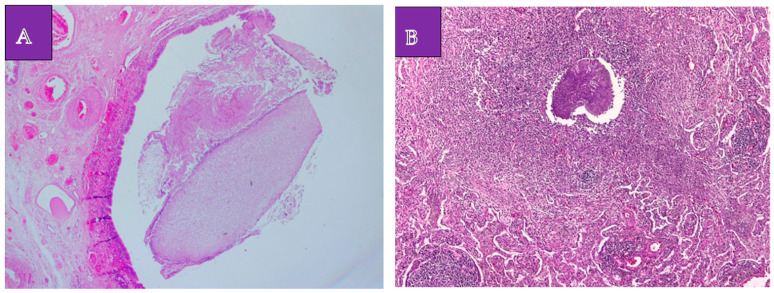
Lung aspergilloma. (**A**). Hematoxylin eosin 50×. Mycotic aggregate (aspergilloma) intrabronchial. (**B**). Hematoxylin eosin 100×. Mycotic aggregation (aspergilloma) in the lung parenchyma associated with acute inflammation.

**Figure 5:**
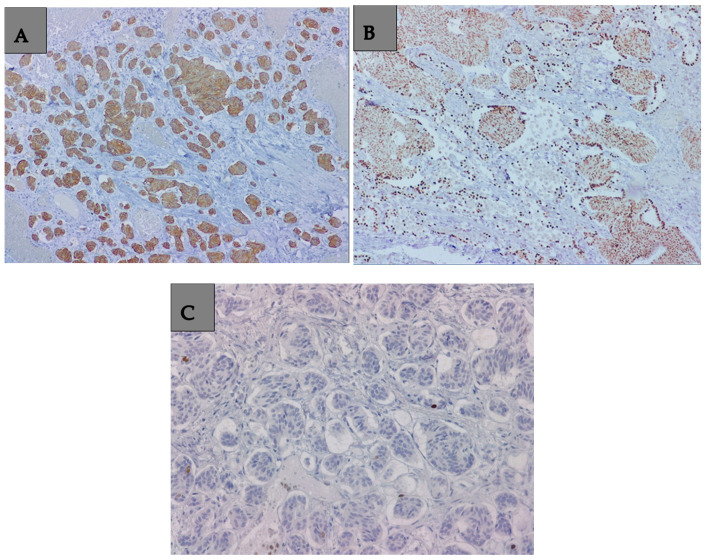
(**A**). Immunohistochemistry image that highlights the positivity of carcinoid tumor cells for synaptophysin. Synaptophysin 100× positive in tumor cells. (**B**). Immunohistochemistry image that highlights the positivity of carcinoid tumor cells for TTF1. TTF1 200× positive at the nuclear level in alveolar epithelial cells and tumor cells. (**C**). Immunohistochemistry image that highlights the positivity of carcinoid tumor cells for Ki67. Ki67 200× positive at the nuclear level in rare tumor cells (1–2 tumor cells).

The histopathological examination established the diagnosis of a lung adenocarcinoma with a non-mucinous lepidic pattern situated in the upper apical segment, low-grade neuroendocrine tumor G1 (a typical carcinoid tumor) at the basal pyramid level (Figure 3A,B), and pulmonary aspergilloma (Figure 4A,B). In the case of the adenocarcinoma, the histological characteristics suggested an in situ/non-invasive lesion. Additionally, immunohistochemistry was performed, with tumor cell positivity for synaptophysin, chromogranin A, TTF1, and Ki67 at a rate of less than 2% (Figure 5A–C). The histopathological examination determined the pT1N0G1 stage of the carcinoid tumor.

The tumor board recommended CT examinations at 6-month intervals during the first 2 years, followed by annual assessments thereafter. In addition, the necessity for antifungal therapy was determined through the recurrent assessment of serum levels of anti-Aspergillus fumigatus antibodies using the hemagglutination method, as well as the Aspergillus galactomannan antigen using the immunoenzymatic method (Table 1). Aspergillus galactomannan antigen values that consistently remained negative ruled out invasive aspergillosis. At this stage, the only decision was to monitor the patient.

In accordance with the guidelines, the patient underwent six-monthly CT evaluations in the first two years; however, no lesions indicative of tumor recurrence were detected. At present, the patient’s clinical condition is good, and she is able to perform her daily activities independently.

## 3. Discussion

The World Health Organization (WHO) classification divides lung neuroendocrine neoplasms (NENs) into four distinct histological types: typical carcinoid (TC), atypical carcinoid (AC), small-cell lung carcinoma (SCLC), and large-cell neuroendocrine carcinoma (LCNEC) [12,13]. The diagnostic criteria encompass the measurement of mitoses on a 2 mm^2^ surface, the identification of necrosis, and the assessment of various cellular and architectural features that delineate the neuroendocrine morphology [13]. Immunohistochemistry plays a crucial role in distinguishing between the four forms of neuroendocrine neoplasms (NENs), namely:

- Well-differentiated tumors of low grade (G1)—TC;
- Well-differentiated tumors of intermediate grade (G2)—AC;
- Poorly differentiated tumors of high grade (G3)—SCLC and LCNEC [14].

The assessment of patients’ prognosis is significantly influenced by the histological classification. Therefore, while the prognosis of TC is favorable, characterized by a good life expectancy, the prognosis of AC is worse because of a higher rate of distant metastasis [15,16]. Furthermore, individuals with SCLC or LCNEC have the highest risk of disease progression, with a shorter life expectancy despite advances in therapeutic management [15,16].

Pulmonary carcinoid tumors are malignant tumors characterized by neuroendocrine differentiation and morphology; they originate from the mature cells of the diffuse pulmonary neuroendocrine system [17]. The latest WHO classification defines TC as tumors with less than 2 mitoses per 2 mm^2^, with no necrotic lesions, and AC as tumors with 2–9 mitoses per 2 mm^2^, coagulation necrosis, or both [18]. In terms of prevalence, the TC:AC ratio is between 8:1 and 10:1 [1]. In a recent investigation, Yang et al. found that young patients and smokers had a higher incidence of AC compared to TC [19]. Our study confirms these findings by diagnosing a TC in an older, non-smoking patient. Aydin et al. reported that the average age of TC patients was 50 years [20]. Under these circumstances, our patient’s elderly age of 75 years can be considered a clinical peculiarity. In terms of symptomatology, most individuals diagnosed with carcinoid tumors exhibit notable symptoms, including cough (59.1%), hemoptysis (29.5%), and fever (18.2%) [19]. The symptoms presented by our patient were cough and hemoptysis.

The co-occurrence of carcinoid tumors and lung adenocarcinoma is extremely rare. Two of these cases were recently documented in the literature by [2,21,22]. The first case is a 77-year-old, non-smoking female with a history of parathyroid adenocarcinoma, which associates with an AC and a papillary adenocarcinoma of the upper lobe of the right lung [2]. The second example is an 83-year-old female smoker with two tumors with different histologies: a TC and a papillary adenocarcinoma [2]. While smoking has been associated with an increased risk of developing multiple primary tumors, it is worth noting that one of these two patients, like our patient, was a non-smoker [2,23].

Another important particularity in our case is the early diagnosis of both tumors. Abbi et al. reported a similar case [24]. These authors highlight the challenge associated with diagnosing such cases and underscore the necessity for extensive resections or lobectomy in order to conduct an accurate histological evaluation [24]. Nagamatsu et al. described a case with two distinct types of tumor cells within the same tumor, a carcinoid component found in the core fibrous scar of an adenocarcinoma [25]. In contrast to this example, our patient exhibited two distinct tumors characterized by varied histological characteristics, situated at a considerable distance from one another. There are two main types of synchronous tumors: collision tumors and composite tumors [26]. Composite tumors are characterized by their shared progenitor cell origin and similar mutational status [26]. Collision tumors arise from distinct progenitor cells and possess a distinct mutational status [26]. Therefore, the case documented by Nagamatsu et al. pertains to composite tumors, whereas our case corresponds to collision tumors. Regarding the pathogenesis of collision tumors, one hypothesis suggests that paracrine signaling arising from an adenocarcinoma may modify the microenvironment, leading to neuroendocrine cell hyperplasia and, therefore, to its malignant transformation [2]. A different hypothesis suggests that carcinoid tumor cells and adenocarcinoma cells originate from similar precursor epithelial cells, wherein further mutations, such as those in the EGFR gene, manifest subsequent to differentiation into bronchioloalveolar cells [2]. Additionally, synchronous tumors may originate from distinct precursor cells, and their concurrent emergence is linked to the same risk factors [2].

The most important particularity of our case is the association between colonization with Aspergillus and two synchronous tumors. After reviewing the scientific literature, we found several cases of associations between pulmonary aspergillosis and carcinoid tumors, as well as pulmonary aspergillosis and pulmonary adenocarcinoma [27,28,29,30,31]. However, we have not discovered any cases that linked all three diseases. The coexistence of a malignant lung tumor and fungal pneumonia presents diagnostic challenges due to the possibility of their simultaneous or consecutive occurrence [30]. Patients with a history of lung cancer are more likely to acquire fungal infections, particularly if they received chemotherapy or radiotherapy [30]. This, nevertheless, does not apply to our patient, as she did not have a history of radiotherapy or chemotherapy. Sometimes, fungal infections may occur prior to the diagnosis of lung cancer, resulting in treatment delays [30]. Pulmonary aspergillosis may have been the initial pathological manifestation in our patient; however, fortunately, it did not impede the timely detection of malignancy. Therefore, an additional characteristic of our case, in addition to the correlation between two histologically distinct tumors and the colonization with Aspergillus, is the early detection of these tumors. Negative anti-Aspergillus antibodies may indicate colonization by non-fumigatus species, although this was not the case in our patient [32]. A study conducted by Hunter et al., in which they examined a cohort of 167 patients diagnosed with chronic pulmonary aspergillosis (CPA), reported the potential occurrence of false-negative outcomes for anti-Aspergillus antibodies in individuals with numerous immunological abnormalities [33]. This could be the situation for our patient, who had two malignant tumors and possibly multiple immunological abnormalities. The possibility that a patient with pulmonary aspergillosis, in addition to certain malignant diseases, will have negative serological tests for this infection may complicate the diagnosis.

This clinical case emphasizes the necessity for further investigations that seek to clarify the mechanisms underlying the pathogenic connection among these three distinct diseases. The ultimate goal is to improve lung cancer patients’ prognosis by implementing more effective monitoring of individuals with risk factors for this malignancy as well as discovering novel therapeutic targets. Lung adenocarcinoma continues to be a highly aggressive malignancy characterized by significant mortality rates. Through this clinical case, we also highlight the possibility of false-negative results for serum tests that can contribute to establishing the diagnosis of pulmonary aspergilloma in patients with various malignant diseases. Furthermore, we question whether lung colonization with Aspergillus contributed to the onset of lung oncogenesis or was secondary to it. We also propose a hypothesis that suggests a potential correlation between the slow growth of both lung cancers, the potential for early diagnosis of lung cancer, and a fungal infection with Aspergillus.

## 4. Conclusions

We reported the first case with the diagnosis of a lung adenocarcinoma with a lepidic pattern, a TC, and local Aspergillus colonization. One distinctive feature of our case is the early detection of both tumors as well as the absence of serum antibodies against Aspergillus, despite the histological identification of this microorganism. These negative results can be attributed to various immunological abnormalities caused by the accompanying malignant diseases. Furthermore, it is important to underscore the need to consider several differential diagnoses when evaluating a patient with persistent symptoms. Further research is required to clarify the pathogenic relationship among these three distinct disorders.

## Figures and Tables

**Table 1:** Serum values of anti-Aspergillus fumigatus antibodies and Aspergillus galactomannan antigen.

Biomarkers	Values	Reference Interval
Anti-Aspergillus fumigatus antibodies	<1:80	<1:320
Aspergillus galactomannan	0.06	<0.5

## Data Availability

Data are contained within the article.
